# MoS_2_ Nanosheets Sensitized with Quantum Dots for Room-Temperature Gas Sensors

**DOI:** 10.1007/s40820-020-0394-6

**Published:** 2020-02-19

**Authors:** Jingyao Liu, Zhixiang Hu, Yuzhu Zhang, Hua-Yao Li, Naibo Gao, Zhilai Tian, Licheng Zhou, Baohui Zhang, Jiang Tang, Jianbing Zhang, Fei Yi, Huan Liu

**Affiliations:** School of Optical and Electronic Information, Wuhan National Laboratory for Optoelectronics, Huazhong University of Science and Technology, 1037 Luoyu Road, Wuhan, 430074 People’s Republic of China

**Keywords:** Gas sensor, Room temperature, Molybdenum disulfide, Quantum dot, Nitrogen dioxide

## Abstract

The Internet of things for environment monitoring requires high performance with low power-consumption gas sensors which could be easily integrated into large-scale sensor network. While semiconductor gas sensors have many advantages such as excellent sensitivity and low cost, their application is limited by their high operating temperature. Two-dimensional (2D) layered materials, typically molybdenum disulfide (MoS_2_) nanosheets, are emerging as promising gas-sensing materials candidates owing to their abundant edge sites and high in-plane carrier mobility. This work aims to overcome the sluggish and weak response as well as incomplete recovery of MoS_2_ gas sensors at room temperature by sensitizing MoS_2_ nanosheets with PbS quantum dots (QDs). The huge amount of surface dangling bonds of QDs enables them to be ideal receptors for gas molecules. The sensitized MoS_2_ gas sensor exhibited fast and recoverable response when operated at room temperature, and the limit of NO_2_ detection was estimated to be 94 ppb. The strategy of sensitizing 2D nanosheets with sensitive QD receptors may enhance receptor and transducer functions as well as the utility factor that determine the sensor performance, offering a powerful new degree of freedom to the surface and interface engineering of semiconductor gas sensors.

**Electronic supplementary material:**

The online version of this article (10.1007/s40820-020-0394-6) contains supplementary material, which is available to authorized users.

## Highlights

- Highly sensitive and selective room-temperature NO_2_ gas sensors by sensitizing MoS_2_ nanosheets with PbS quantum dots were demonstrated. In this device architecture, the receptor and transduction function as well as the utility factor of semiconductor gas sensors could be enhanced simultaneously.
- The strategy of sensitizing 2D semiconductors with quantum dots as sensitive and selective receptors for gas molecules may offer a powerful new degree of freedom to the surface and interface engineering of semiconductor gas sensors.

**Electronic supplementary material:**

The online version of this article (10.1007/s40820-020-0394-6) contains supplementary material, which is available to authorized users.

## Graphical abstract

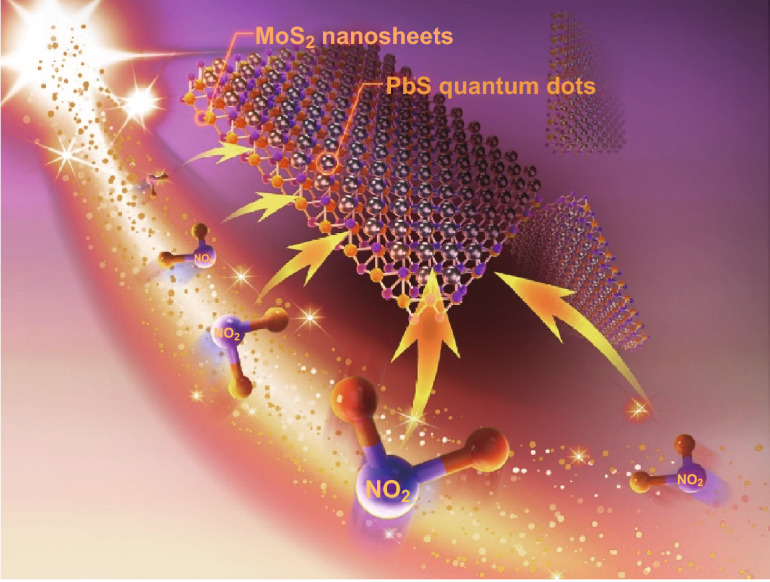

## Introduction

Hazardous air pollutants have become a serious problem for the ecosystem and public health [1, 2]. Nitrogen dioxide (NO_2_) primarily gets in the air from the burning of fuel. Exposure to NO_2_ may potentially increase susceptibility to respiratory infections, and a 5-min emergency exposure limit of 35 ppm NO_2_ exposure has been proposed by the American Industrial Hygiene Association [1, 3]. The large-scale networking of gas sensors for achieving online NO_2_ monitoring requires the power consumption of the sensors to be lower. While semiconductor gas sensor have been widely used in home alarm system owing to their high sensitivity, simple operation, and low cost [4–6], their scale-up application in environmental internet has not been achieved due to the limitation of high operating temperature (typically above 300 °C) which raises the power consumption. The high operating temperature of semiconductor gas sensors also sets a limit to their integrability with CMOS technology or flexible electronic system. Thereby, novel nanostructured materials [7–10] with the potentials for room-temperature gas sensors have become a hot research topic.

MoS_2_ is a well-known 2D graphene-like transition metal dichalcogenides (TMDs). With relatively high carrier mobility, large surface-to-volume ratio, and abundant edge sites which can provide active adsorption sites for gas molecules [11–14], MoS_2_ has been demonstrated as one of the promising materials candidates for room-temperature NO_2_ gas sensors. Liu et al. [13] reported CVD growth of monolayer MoS_2_ for room-temperature detection of NO_2_ with a response time of several minutes without a full recovery to the initial state. Cho et al. [15] demonstrated a charge-transfer-based sensitive NO_2_ gas sensor by CVD-synthesized atomic-layered MoS_2_, with a sensitivity of 220% and a long time (more than 30 min) to recovery. Similarly, chemical exfoliated MoS_2_ prepared by Jung et al. had an incompletable recovery to NO_2_ at room temperature [16]. Kumar et al. fabricated a high-performance NO_2_ sensor based on MoS_2_ with abundant active edge sites. When operated at 60 °C, it had a fast response (16 s) with complete recovery (172 s) with a relative response of 18.1% to 5 ppm NO_2_ [17]. As an alternative strategy, UV light irradiation or gate effect was employed to improve sensitivity toward NO_2_ of MoS_2_ sensor [18–21]. Pham et al. [18] employed LED illumination to improve sensitivity of CVD grown single-layer MoS_2_, achieving sub-ppb limit of NO_2_ gas detection. However, the comparatively high sensitivity and fast response/recovery kinetics at room temperature were not simultaneously obtained for pristine MoS_2_ gas sensors. They suffer from the trade-off between receptor and transducer function. For semiconductor gas sensors, the structural defects are always necessary for gas molecule reception and, on the contrary, may decrease the electronic transduction.

Recently, MoS_2_-based nanocomposites or hybrids through surface modification with noble metals [11], architecture design of hetero-nanostructures with metal oxide nanoparticles [22, 23], and functionalization with other 2D-layered materials such as graphene [24–28] have been demonstrated with improved sensitivity and fast response/recovery kinetics. Motivated by this strategy, we proposed to improve the room-temperature response and recovery by sensitizing MoS_2_ nanosheets with quantum dots (QDs), a highly tunable zero-dimensional (0D) nanomaterial with size-dependent bandgap and excellent solution processability [29–35]. The huge amount of surface dangling bonds of QDs makes them sensitive receptors for gas molecules. Herein, the PbS QDs-sensitized MoS_2_ nanosheets were obtained via a two-step solution process. The sensor had an excellent response of 6.15, to 10 ppm NO_2_ at room temperature, almost five times greater than that of pristine MoS_2_ nanosheets. The sensing mechanism was attributed to the enhanced receptor and transducer functions as well as the utility factor which determine the performance of semiconductor gas sensors.

## Experimental

### Preparation of MoS_2_ Nanosheets

**Fig. 1:**
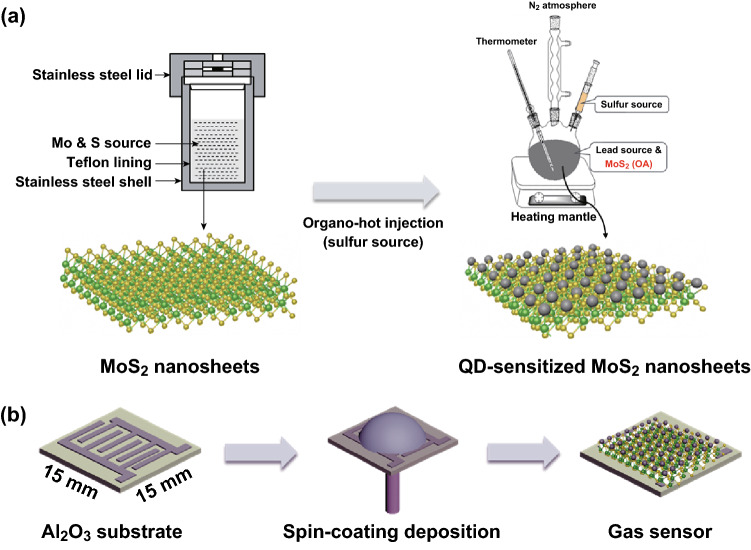
Schematic illustration of **a** formation of the MoS_2_ nanosheets and QD-sensitized MoS_2_ nanosheets and **b** fabricated process diagram of the sensitized structure-based gas sensors

In a typical hydrothermal synthesis of MoS_2_ nanosheets [36], as shown in Fig. 1a, 1 mmol hexaammonium heptamolybdate tetrahydrate ((NH_4_)_6_Mo_7_O_24_·4H_2_O) and 14 mmol thiourea were dissolved into 35 mL of deionized water under stirring for several minutes to form a homogeneous solution. The mixed solution was transferred into a 50-mL Teflon-lined stainless steel autoclave to react at 220 °C for 18 h and then naturally cooled down to room temperature. The final product was rinsed with deionized water and absolute ethanol several times to remove any possible ions. After drying at 70 °C for 6 h, black MoS_2_ nanosheet powder was obtained.

### Synthesis of MoS_2_ Nanosheets Sensitized with QDs

Figure 1a shows the synthesis of MoS_2_ nanosheets sensitized with PbS QDs. Organo-hot injection method has always been proven as an effective method for QD synthesis [37–39]. First, the as-prepared MoS_2_ powders (20 mg) were dissolved in 4 mL of oleic acid (OA). Ultrasonic dispersion was conducted for 30 min to ensure the black powder was completely dispersed in the solution. PbO (2 mmol), OA (2 mmol), 1-octadecene (ODE) (20 mL), and as-prepared MoS_2_ (OA) solution (530 μL) were all mixed in a three-neck flask and heated to 90 °C under a vacuum for 6 h. Then, the reaction temperature was raised to 120 °C and 0.33 mmol bis(trimethylsilyl) sulfide (TMS) mixed with ODE (10 mL) was rapidly injected under an inert atmosphere. The reaction lasted for 30 s, and the mixture was then transferred to cold water bath for rapid cooling to room temperature. The nucleation and growth of QDs anchoring in the surface of MoS_2_ nanosheets occurred in this process. The product was precipitated by acetone and re-dispersed in toluene several times to prepare PbS–MoS_2_ solution for device fabrication.

### Sensor Fabrication

The layer-by-layer spin-coating deposition technique of the sensitized MoS_2_-based thin film was carried out in ambient air at room temperature (a schematic illustration can be seen in Fig. 1b). Alumina ceramic substrates (15 × 15 × 0.8 mm^3^) prepatterned with a pair of interdigital Ag electrode (the spacing and width are 5 mm) were prepared via screen printing. Then 70 μL of PbS–MoS_2_ solution was dropped onto the substrate, which was then spun at 2350 rpm for 30 s. Next, four drops of NaNO_2_ diluted in methanol (10 mg mL^−1^) were added dropwise to the film for ligand exchange, with a wait time of 45 s, and spun dry at 2500 rpm for 30 s, followed by repeating the NaNO_2_ treatment twice. Finally, the film was washed by methanol flush and then spun dry three times to obtain 3-layers thin-film device. The film deposition process was repeated three times. For comparison, the pristine MoS_2_ nanosheet device was prepared according to the following steps. First, the prepared substrates were placed in a hotplate with a heating temperature of 135 °C. Next, a drop of MoS_2_ ethanol solution was deposited dropwise onto the thermal substrate and naturally dried for a few seconds, followed by repeating the process twice. Finally, the fabricated MoS_2_ sensor was maintained under the thermal treatment for 20 min.

### Characterization and Measurements

A field emission scanning electron microscope (FE-SEM, GeminiSEM 300, Zeiss, Oberkochen, Germany) equipped with an energy-dispersive X-ray spectrometer (EDS, X-MAX, Oxford, UK) was used to obtain SEM images and elemental mapping data. Transmission electron microscopy (TEM) images were recorded with a Tecnai G2 20 microscope operating at an accelerating voltage of 200 kV. X-ray diffraction (XRD) measurements were obtained using a diffractometer (Empyrean, PANalytical B. V., Netherlands) with Cu Kα radiation in the 2*θ* range of 10–70 °C. An energy-dispersive X-ray spectrometer (EDS) was performed on a XL 30 ESEM FEG. X-ray photoelectron spectroscope (XPS) measurements were using by an AXIS-ULTRA DLD-600 W with an Al source, and C 1*s* peak at 284.5 eV is used as reference. Similarly, ultraviolet photoelectron spectroscopy (UPS) measurement was also performed by using the same system with a He-Iα 21.22 eV UV light. Work functions were measured by a KP 020 K probe (KP Technology, Wick, Scotland). UV–Vis–NIR absorption spectra were measured using a PerkinElmer Lambda 950 UV–Vis–NIR spectrophotometer.

The NO_2_ sensing measurements were carried out by a computer-connected source meter system (Model Keithley 2450/6487, Keithley Instruments, USA) under static conditions controlled with the relative humidity (RH) being 19–85% at room temperature (sensor setup details as shown in Fig. S1). The sensor response was defined as the ratio of *R*_a_ to *R*_g_, where *R*_a_ is the baseline resistance in the ambient atmosphere and *R*_g_ is the resistance of the sensor device in the presence of NO_2_ gas. The response time (*T*_90_) and the recovery time (*T*_10_) were defined as the time taken by the sensor response to reach 90% of its maximum value upon exposure to NO_2_ gas and drop to within 10% of its original baseline value after removal of gas.

## Results and Discussion

### Structural Properties of MoS_2_ Nanosheets and QD-Sensitized MoS_2_ Nanosheets

**Fig. 2:**
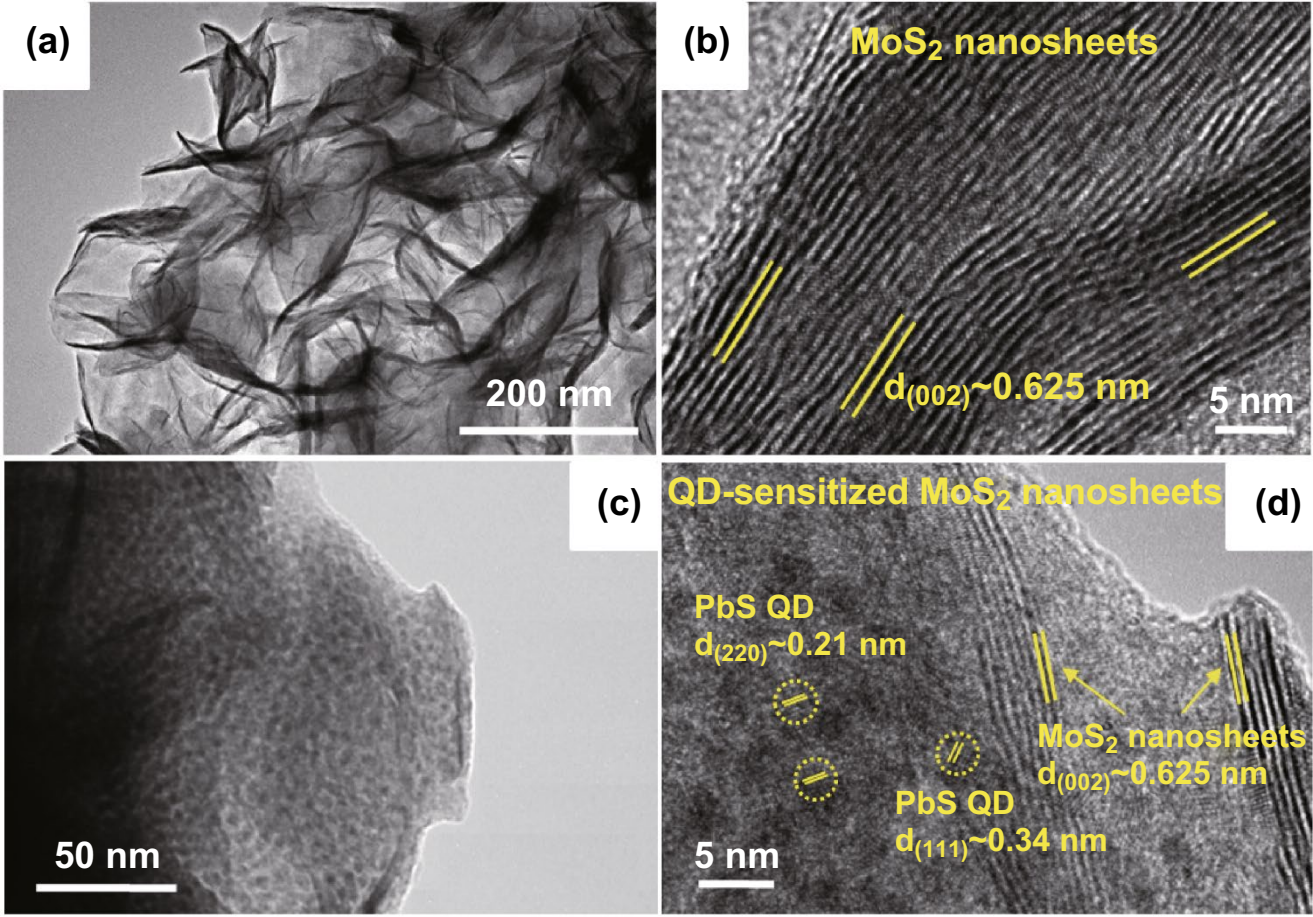
Morphology of the MoS_2_ nanosheets and QD-sensitized MoS_2_ nanosheets: **a**, **b** TEM images of the flowerlike MoS_2_ nanosheets and **c**, **d** QD-sensitized MoS_2_ nanosheets at different magnifications, showing a lattice space of 0.625 nm corresponding to the (002) lattice plane of MoS_2_, and 0.21, 0.34 nm corresponding to the (220), (111) lattice planes of PbS, respectively

The morphology of the MoS_2_ and QD-sensitized MoS_2_ nanosheets was characterized with SEM and TEM, respectively. Figure 2a displays a low-magnification TEM image of MoS_2_ nanosheets revealing the ultrathin nanosheet morphology with slightly assembly character. Further, more lattice fringes were clearly indicated from high-magnification TEM image (Fig. 2b), revealing the labeled lattice spacing of 0.625 nm, which was in a good agreement with the (002) lattice plane with MoS_2_ nanosheets. The abundant MoS_2_ nanosheets layers provide large quantities of edge sites, which may beneficial for gas molecules absorption. Moreover, Fig. S2 shows an SEM image of the as-prepared MoS_2_ nanosheets distributed on the alumina ceramic substrate, and the observable flowerlike MoS_2_ nanosheets were uniformly assembled by a mass of bent flakes. Similarly, TEM images of different magnifications in Fig. 2c, d used to observe more detailed microstructure information of the QD-sensitized MoS_2_ nanosheets. A large amount of QDs formed on the edge sites of the MoS_2_ nanosheets as demonstrated in Fig. 2c. This could be attributed to the edge area defects, which provide more active sites for the nucleation of PbS QDs. Pb atoms can fill the vacancy on the MoS_2_ surface, which may weaken the MoS_2_ defects [40]. Equally important is that the MoS_2_ surface might be spontaneously functionalized with the excessive OA molecules in the reaction process, and then the strong hydrophobic interaction [41, 42] of the OA ligands on both the QDs and MoS_2_ surfaces leading to the noncovalent binding of QDs to MoS_2_. However, the detailed mechanisms regarding how the OA ligands or molecules take part in the synthesis of MoS_2_ nanosheets sensitized with QDs need further investigation. The efficient attachment and coverage of the QDs onto the MoS_2_ nanosheets are further indicated by the high-resolution TEM image in Fig. 2d. Well-crystallized QDs with diameters of approximately 3.26 nm were uniformly separated on the surface of the MoS_2_ nanosheets. The lattice spacings of these spherically shaped QDs were 0.21 and 0.34 nm, corresponding to the (220) and (111) lattice planes of PbS, respectively. The edges of the MoS_2_ nanosheets were not continuous, probably because some defects were generated in the synthesis processes. The typical elemental mapping data were characterized by EDS, as shown in Fig. S3a–e, which also confirmed the even distribution of the Pb and Mo element in the final actual device, revealing the formation of well-distributed PbS QDs in the MoS_2_ nanosheets.

**Fig. 3:**
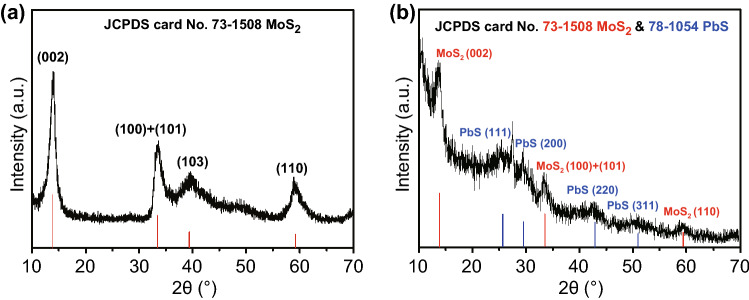
XRD patterns of **a** MoS_2_ nanosheet and **b** QD-sensitized MoS_2_ nanosheets

To further confirm the structural information of the MoS_2_ and QD-sensitized MoS_2_, the XRD patterns of the samples are shown in Fig. 3. It indicates that the four sharp diffraction peaks centered at approximately 2*θ *= 13.9°, 33.4°, 39.4°, and 58.9° of the powder MoS_2_ could be well-indexed, respectively, to the (002), (100) + (101), (103), and (110) planes of the hexagonal phase MoS_2_ (JCPDS card No. 73-1508). The strong (002) peak at 2*θ *= 13.9° with a d-spacing of approximately 0.625 nm corresponded to a well-stacked layered structure along the c axis as well as the TEM results. Compared to the pristine MoS_2_, the XRD patterns of the sensitized structure in Fig. 3b contained some extra peaks other than the main characteristic peaks of MoS_2_. The peaks at approximately 2*θ *= 25.3°, 29.6°, 42.8°, and 51.4° were not only well matched with the (111), (200), (220), and (311) planes of cubic PbS (JCPDS card No. 78-1054), which indicated the successful growth of PbS QDs on the surface of MoS_2_ nanosheet, but also consistent with the TEM characteristics presented in Fig. 2d. The significantly broadened peak that appeared on PbS could possibly be attributed to the quantum size feature of the QDs, according to the Debye–Scherrer equation.

The surface elements and chemical states of the sensitized MoS_2_-based film were characterized by X-ray photoelectron spectroscopy (XPS) in the supporting information. As expected, Pb, Mo, and S were detected on the film, which was consistent with the EDS results. Figure S4a–c shows the high-resolution XPS spectra of Pb 4f, S 2p, and Mo 3d, respectively. Two peaks located at 142.7 and 137.8 eV correspond to the 4f_5/2_ and 4f_7/2_ of the Pb^2+^ state exhibited in Fig. S4a. Most of the Mo signal is from its Mo^4+^ state at the peak positions around 228.5 and 229.2 eV, mainly corresponding to Mo^4+^ 3d_5/2_ (Fig. S4c). Two dominant S 2*p* peaks were observed around 161.5 and 162.2 eV (Fig. S4b), accompanied by a slightly flat peak at 163.8 eV, which were assigned to the divalent sulfide ions (S^2−^) of the MoS_2_ and PbS.

### NO_2_ Gas-Sensing Properties

**Fig. 4:**
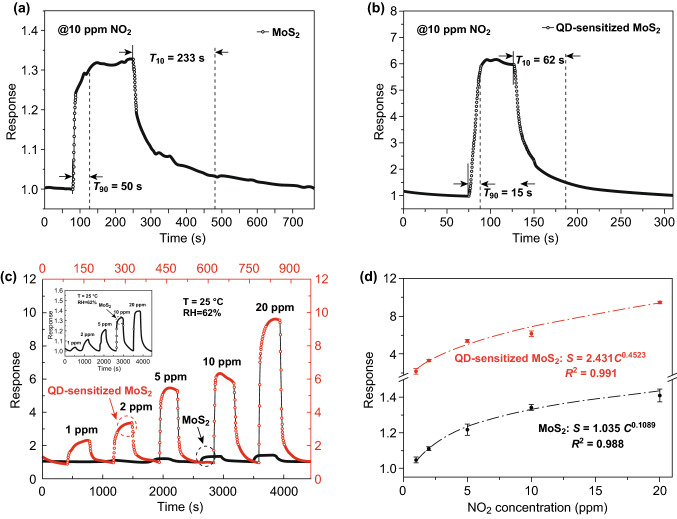
Time-resolved response and recovery curves of **a** MoS_2_ nanosheets and **b** the sensitized MoS_2_ gas sensors exposed to 10 ppm NO_2_ at room temperature. **c** Transient relative response of both sensors toward different NO_2_ concentrations. **d** The relative response versus the NO_2_ concentration illustration of the MoS_2_ and the sensitized MoS_2_ sensors

The NO_2_-sensing performance was measured using a homemade computer-connected source meter system under room temperature. We performed repeatability test for the both devices at the same time and measured the relative response to six and four successive cycles toward 10 ppm NO_2_ for pristine MoS_2_ nanosheets and the sensitized MoS_2_ gas sensors, respectively (Fig. S5a). The pristine MoS_2_ sensor showed the complete recovery at room temperature without any extra stimulus such as optical or thermal source; however, the completed response/recovery cycle required a slightly time. After sensitization by the PbS QDs, the sensitized MoS_2_ sensor exhibited an obviously enhanced response to the same concentration of NO_2_ gas, also with a fast response/recovery time and excellent reversibility. Transient resistance characteristic of MoS_2_ nanosheets and the sensitized MoS_2_ gas sensors to 10 ppm NO_2_ is shown in Fig. S5b, exhibiting p-type gas-sensing behavior for both sensors. The improved performance can be attributed to the excellent access of gas molecules adsorption by the PbS QDs as NO_2_ receptors, as well as the favorable 0D-2D interface for charge transfer, which will be discussed in detail later. Three kinds of theoretical Mo to Pb molar ratio (2%, 5%, and 8%) were used in the precursor solutions during the synthesis, and we found that sensor response was much higher by a medium molar ratio of 5% (Fig. S6). Thus, we used this optimal molar ratio to sensor fabrication in this work. The representative time-resolved response and recovery curves of the pristine MoS_2_ and the sensitized MoS_2_ gas sensor were illustrated in more detail in Fig. 4a, b. In general, many defects may occur in the surface of MoS_2_, which can lead to a strong chemisorption between MoS_2_ and gas molecules, so that NO_2_ or other gases such as O_2_ are difficult to desorb from the MoS_2_ [43], resulting in a weakened recovery kinetics, as shown in Fig. 4a. The sensitized MoS_2_ sensor exhibited a superior performance not only with an excellent response of 6.15 to 10 ppm NO_2_, which was almost five times greater than the pristine MoS_2_ device, but also with an outstanding response/recovery ability, with the time improving from 50/233 to 15/62 s, respectively.

**Fig. 5:**
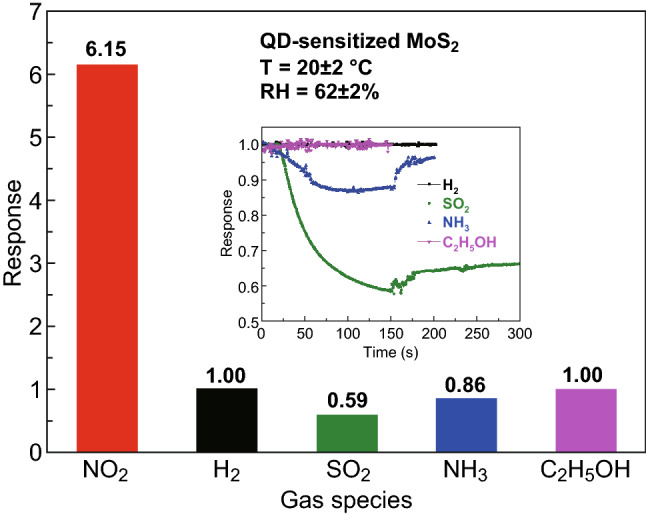
Selectivity of QD-sensitized MoS_2_ gas sensors toward different gases: 10 ppm NO_2_, H_2_, SO_2_, NH_3_ and 200 ppm C_2_H_5_OH

To further investigate the NO_2_-sensing properties of the sensors, the dynamic response curves were recorded with the NO_2_ concentration of 1, 2, 5, 10, and 20 ppm, respectively, shown in Fig. 4c. Both devices showed recoverable response under room temperature, and the response values gradually increased with the increasing NO_2_ gas concentration. Obviously, the device based on MoS_2_ nanosheets sensitized with QDs was more sensitive than the pristine MoS_2_ for NO_2_ gas detection and indicated potential for a lower limit of detection (*LOD*). The pristine MoS_2_ had less of a response when exposed to 1 ppm NO_2_, while the sensitized MoS_2_ sensor still performed 2.30 toward the same concentration with a rapid response/recovery rate, which even better than the measurement to 20 ppm of pristine MoS_2_ device (details are shown in Fig. S7). Owing to this improvement, the theoretical *LOD* for NO_2_ was calculated to be 174 and 94 ppb in the case of pristine MoS_2_ and QD-sensitized MoS_2_, respectively (calculation details in Fig. S8). However, the measurement error of the *LOD* for both sensors is mainly from accuracy of gas concentration and errors in test results. The dependence of the sensor response on gas concentrations range from 1 to 20 ppm is also analyzed in Fig. 4d. The fitting equation between the response value (*S*) and NO_2_ concentration (*C*) can be illustrated as a power law relationship, and the exponent was estimated to be 0.1089 together with a coefficient of determination (*R*^2^) value of 0.988 for the MoS_2_ sensor, while the values were 0.4523 and 0.991 for the sensitized MoS_2_ sensor. Importantly, the theoretical analysis of the relationship between the response values and gas concentrations was significant for the gas sensor, which will facilitate the determination of gas concentrations in practical applications. Selectivity is considered as an important parameter for gas sensors, and we compared the response of the sensitized MoS_2_ gas sensors toward several gases in our lab. As shown in Fig. 5, the sensors exhibited high response to 10 ppm NO_2_ gas and negligible response to 10 ppm H_2_, SO_2_, NH_3_ and 200 ppm C_2_H_5_OH vapor, respectively, at room temperature. The inset showed the dynamic response curves upon gas exposure and release of the intervening gases, respectively. We also investigated the NO_2_-sensing performance of the sensitized MoS_2_ sensors in the range of RH of 19%, 29%, 48%, 65%, and 85%. The sensor response toward 10 ppm NO_2_ had a tendency to grow over the RH gradually increased (shown in Fig. S9a). While the functional relationship between relative humidity and response could be further defined clearly, we can use humidity compensation methods to make our sensors satisfy the practical application under environment with a wider range of the RH. More details are shown in Fig. S9b about the real-time sensing curves toward 10 ppm NO_2_ at different RH based on the sensitized MoS_2_ gas sensors, revealing fast response/recovery kinetics under any RH environments. For this specific investigation, the RH value was intentionally controlled at certain values with an accuracy of 2%. The average sensitivity to 10 ppm NO_2_ under RH ~ 65% was 6.19, which was close to the average sensitivity of 6.14 under RH ~ 62% (Fig. 4d). Therefore, the RH ranged from 62 to 65% was within the error range. Under high RH environments, we suspected that water molecules preadsorbed on the surface of the sensitized MoS_2_, dissociating into OH^−^ and H^+^ to form hydroxyl groups. Hydroxyl groups as an electron donor lead to increase in resistance of the materials [44]. NO_2_ has strong adsorption properties compared with the physical adsorption of water molecules. When NO_2_ injected, they could kick out the physical adsorption of water molecules and cause a further decrease in resistance, thus achieving a higher response. Actually, it is reported that the hydroxyl groups could improve the NO_2_-sensing performance in recent study [35, 45–47].

Compared to other MoS_2_-based NO_2_ sensors (Table 1), our MoS_2_ nanosheets-based sensor only maintained a general level at room-temperature (RT) operation; however, under the same conditions, the sensitized MoS_2_ sensor had a superior performance with no thermal treatment or UV illumination [21, 48]. Compared to the most MoS_2_-based gas sensors in the current published papers, the sensitized MoS_2_ gas sensor exhibited an excellent response from 6.15 to 10 ppm NO_2_ at room temperature, accompanied by a rapid response/recovery time of 15/62 s, indicating high sensitivity and outstanding recovery ability. In addition, as shown in Fig. S9c, long-term stability test of the sensitized MoS_2_ sensor upon 10 ppm NO_2_ was consistent with the effect of relative humidity on the sensing performance. Furthermore, QD-sensitized MoS_2_ nanosheets with excellent solution processability are particularly attractive for next-generation gas sensors compatible with silicon-based or flexible substrates.

**Table 1:** NO_2_-sensing performance of MoS_2_-based sensors

Materials	Method	Work temperature (°C)	Concentration (ppm)	Response (%)	*T*_90_/*T*_10_ (s)	References
Few-layer MoS_2_	Mechanically exfoliating	RT	100	60	180/600	[12]
Monolayer MoS_2_	CVD	RT	0.4	80	~ 420/-(incomplete)	[13]
Few-layer MoS_2_	CVD	RT	10	60	~ 60/~ 1000	[14]
Atomic-layered MoS_2_	CVD	RT	1.2	150	~ 60/~ 1800	[15]
MoS_2_ nanowires	CVD	60	5	18.1	16/172	[17]
Single-layer MoS_2_	CVD	RT with LED light	0.1	~6	~ 500/~ 1	[18]
Multilayer MoS_2_	Mechanically exfoliating	RT with gate effect	100	4	~ 60/~ 60	[19]
Multilayer MoS_2_	CVD	RT with UV light	100	35	29/350	[48]
Mixed MoS_2_ flakes	CVD	RT with UV light	10	21.78	6.09/146.49	[21]
SnO_2_ NC-MoS_2_ NS	Chemical exfoliation	RT	10	28	400/180	[22]
ZnO NPs/MoS_2_ NSs	Wet chemical method	RT	5	3050	40/~ 600	[23]
MoS_2_-RGO	Liquid exfoliation and hydrothermal	160	3	129	8/20	[25]
WS_2_ functionalized MoS_2_	Hydrothermal process	RT	50	26.12	1.6/< 30	[26]
MoS_2_ nanosheets	Hydrothermal	RT	10	133	50/233	This work
MoS_2_ nanosheets sensitized with QDs	Hydrothermal and organo-hot injection	RT	10	615	15/62	This work

### Gas-Sensing Mechanisms

**Fig. 6:**
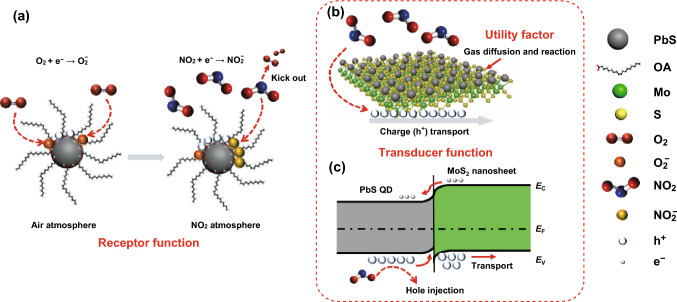
Schematic illustration of the NO_2_-sensing mechanism of MoS_2_ nanosheets sensitized with QDs. **a** Receptor function of PbS QDs. **b** Transducer function of MoS_2_ nanosheets and the utility factor involved for the sensitized MoS_2_ nanosheets. **c** Interface band structure of PbS QD-MoS_2_ nanosheet

As previously noted, the gas sensor based on MoS_2_ nanosheets sensitized with QD had a good NO_2_-sensing performance at room temperature, which was quite possible for the combinational effects between the PbS QDs and MoS_2_ nanosheets. Therefore, we proposed three basic factors of receptor function, transducer function and utility [49], as well as an interface energy band diagram to investigate the sensing mechanism of QD-sensitized MoS_2_ nanosheets. As illustrated in Fig. 6a, PbS QDs always exhibited p-type conduction behavior in air atmosphere because of physisorbed O_2_ molecules, which consumed electrons and introduced lots of holes as well. When exposed to NO_2_ gas, according to our previous research [32, 34, 35], due to the strong binding energy compared to O_2_, NO_2_ kicks out the originally physisorbed O_2_ molecules and binds to Pb^2+^ through O, introducing more charge-transfer-driven *p*-type doping and developing a hole concentration in the *p*-type PbS QDs. For pristine *p*-type MoS_2_ nanosheets, the defects mainly on the edge sites of the MoS_2_ acted as active sites for NO_2_ molecules, and these defects dominated process contributing to the poor response, slow rates of response, or even incomplete recovery due to high energy binding sites [50], especially operation at room temperature without any illumination. Thus, the inevitable receptor–transducer function [51] conflict cannot be well addressed in the pristine MoS_2_-based gas sensor. After sensitization with QDs (illustrated in Fig. 6b), most of the high energy binding sites on the surface of MoS_2_ were occupied by the highly active QD receptors which had larger surface-to-volume ratio as well as abundant surface defects (mainly from dangling bonds, surface Pb sites, sulfur vacancies, etc.) capable of active interaction with NO_2_ gas molecules adsorption, contributing to a marked enhancement in the response. Furthermore, the adsorption energies of NO_2_ on the MoS_2_ and PbS were calculated based on the density functional theory (DFT) in the previous literature, indicating that the adsorption energy of NO_2_ on the PbS is significantly larger than that on MoS_2_ [52]. Therefore, PbS QDs may serve as receptors of NO_2_ molecules and enhance the receptor function of the MoS_2_ sensors.

Combining with Fig. 6b, we also used an interface energy band diagram to further study the sensing mechanism. To simulate the actual environment, Kelvin probe measurement was carried out in ambient air. The work function (*W*_F_) of the PbS QD and MoS_2_ nanosheet was approximately 4.61 and 4.96 eV, respectively. Next, we used ultraviolet photoelectron spectroscopy (UPS) to confirm the valence-band edge (*E*_v_) [53] and the scan of the spectra for both as shown in Fig. S10. The *E*_v_ value was calculated to be 5.20 and 5.46 eV for the PbS QD and MoS_2_ nanosheet, respectively. We also introduced a UV–Vis–NIR absorption spectrum mainly to evaluate the energy bandgaps (*E*_g_) of the MoS_2_ nanosheet and PbS QD. As shown in the MoS_2_ spectrum in Fig. S11a, the characteristic absorption peaks that appeared in the visible regions were consistent with the general features of TMDs with trigonal prismatic coordination, which confirmed the 2H polytype of the MoS_2_ nanosheet [54]. The intercept was interpolated inside giving the value to *E*_g_ of 1.50 eV for MoS_2_ through the Kubelka–Munk transformed reflectance spectra, indicating that the prepared MoS_2_ with few-layer nanosheets possesses a bandgap larger than the bulk materials. Figure S11b shows that an exciton absorption peak appeared in 992 nm, from which we could obtain the calculated *E*_g_ of 1.25 eV of PbS QD. It exhibited a significantly broadened bandgap compared to the bulk PbS (0.41 eV), confirming a conservation of strong quantum confinement effect [55]. Taking together the above experimental parameters, the initial condition (before mutual contact) of the energy band structure for PbS QD and MoS_2_ nanosheet could be illustrated in Fig. S12. Because of the difference in work functions (4.61 vs. 4.96 eV), when the PbS and the MoS_2_ were brought into contact, the electrons pass from the PbS to MoS_2_, creating a positive charge region closed to the PbS surface and opposite one near the MoS_2_ surface. Finally, interface band structure was developed for both sides as band bending occurred and a potential barrier of 0.35 eV $\left( {\varphi_{\text{F}} = W_{{{\text{F}}({\text{PbS}})}} - W_{{{\text{F}}({\text{MoS}}_{2} )}} } \right)$ formed in the contact position, which was accompanied by the balanced *E*_F_. As exhibited in the diagram in Fig. 6c, a majority of the NO_2_ molecules adsorbed on the surface of the QD receptors may form donor-like surface states in general, and a direct electron extraction from the conduction band of QD into the NO_2_ molecules, which also meant hole injection from the NO_2_ into the valence band of QD. Anyway, a mass of holes will accumulate at the interface closed to the side of the PbS QDs during its receptor function process. Equally important was that the MoS_2_ nanosheets served as the conductive path in the system, leading the NO_2_-induced holes flow to the electrode for collection, easily overcoming the relatively low potential barrier generated at the interface of the valence-band edge. DFT calculation results recently demonstrated that the diffusion barrier is only dozens of meV for NO_2_ on MoS_2_, which also proved that NO_2_ gas molecules may easily diffuse rapidly on MoS_2_ surface [40]. Thus, MoS_2_ nanosheets can serve as the charge transport highway for the effective transducer function of the sensitized surface adsorption of NO_2_ gas molecules into an electrical resistance change of the sensor.

Concluded from the above discussion, the sensitized MoS_2_ sensor had a good response and recovery kinetics even at room temperature because of the favorable 0D QD-2D MoS_2_ interface, combining the improvement of both receptor function and transducer function [49, 51, 56]. Beyond that, the utility factor is one of the important factors which concerns the gas-sensing performance and goes up with the smaller pore size as well as thinner gas-sensitive film [49]. We took characterization about SEM cross-section morphology of the sensitized MoS_2_ based on alumina ceramic substrate. However, it was difficult to observe the thickness of such nanothin film clearly on the rough ceramic substrate because it was hard for cutting. Hence, we employed the comparative smooth silicon substrate for material deposition. Figure S13a displays the cross section of the three-layer QD-sensitized MoS_2_ thin film on silicon substrate, revealing a conformal film deposition, and the film thickness was estimated to be 135 nm. Thus, the utility factor could be benefited greatly from the relatively porous thin-film features, which enhanced the accessibility of inner sulfide grains to the NO_2_ molecules, leading to enhanced gas diffusion and reaction, thereby achieving higher response along with shorter response/recovery time. We further provided more details in Fig. S13b about NO_2_-sensing performance of different deposited layers and finally found that the three-layer thin-film-based sensors had a stable response together with a fast recovery time. In brief, our sensitized MoS_2_ gas sensors exhibited a better NO_2_ gas-sensing performance at room temperature than that of the pristine MoS_2_ sensors. The sensitized MoS_2_ architecture overcome the receptor–transducer function conflict limitation, as well as enhanced the utility factor by sensitizing MoS_2_ nanosheets with QDs. More importantly, a deeper understanding of the 0D-QDs with tunable bandgaps will further promote progress in the engineering of energy band alignment at the 0D-2D heterojunction interface, paving a promising way to develop gas-sensing performance of 2D layered materials.

## Conclusions

In summary, we proposed a facile synthesis strategy for sensitizing MoS_2_ nanosheets with PbS quantum dots as NO_2_ gas molecules. The sensitized MoS_2_ gas sensor exhibited sensitive and recoverable response at room temperature, with the response/recovery time shortened from 50/233 to 15/62 s upon 10 ppm of NO_2_ exposure/release cycle, respectively, compared to the pristine MoS_2_ nanosheets. The gas-sensing mechanism was attributed to the fundamental factors of receptor function, transducer function and utility, as well as the favorable 0D-2D interface between QDs and MoS_2_ nanosheets. Through the surface sensitization of MoS_2_ nanosheets with PbS QDs as sensitive and selective NO_2_ receptors, combined with the favorable charge transfer at interfaces and excellent charge transport, the receptor and transducer function as well as the utility factor were desirable enhanced, thereby achieving the enhanced performance for NO_2_ gas sensing. This work demonstrated a novel sensitized MoS_2_ gas sensor with superb sensitivity and extremely low power consumption. The solution-processable and room-temperature operable gas sensors could be integrated with silicon-based or even flexible substrates to achieve smart on-chip electronic nose.

## Supplementary Material

Homemade sensor setup; SEM image of the flowerlike MoS_2_ nanosheets; EDS elemental mapping of QD-sensitized MoS_2_ nanosheets; XPS characterization of QD-sensitized MoS_2_ nanosheets; repeatability curves and transient resistance characteristic of the MoS_2_ nanosheets and QD-sensitized MoS_2_ nanosheets sensors; sensor response of QD-sensitized MoS_2_ with different Pb:Mo; transient relative response of MoS_2_ sensors toward different NO_2_ concentrations; LOD calculation of MoS_2_ nanosheets sensor and QD-sensitized MoS_2_ sensor; sensor response at different relative humidity and long-term stability of the QD-sensitized MoS_2_ gas sensors; UPS characterization of MoS_2_ nanosheets and PbS QDs; UV–Vis–NIR spectra of MoS_2_ nanosheets and PbS QDs; the initial energy band structure of PbS QD and MoS_2_ nanosheet; SEM cross-section morphology of QD-sensitized MoS_2_ thin film; and NO_2_-sensing properties of QD-sensitized MoS_2_ with different deposition layers.

## Electronic supplementary material

Below is the link to the electronic supplementary material.

Supplementary material 1 (PDF 1475 kb)
