# Supplementary material for: MoS2 Nanosheets Sensitized with Quantum Dots for Room-Temperature Gas Sensors
Source: Nanomicro Lett. 2020 Feb 19;12:59. doi: 10.1007/s40820-020-0394-6 (PMC7770826; doi:10.1007/s40820-020-0394-6)
Supplement: Supplementary file 1 — Supplementary material 1 (PDF 1475 kb) [file 40820_2020_394_MOESM1_ESM.pdf]

Supporting Information for

MoS_2 Nanosheets Sensitized with Quantum Dots for Room-Temperature Gas Sensors

Jingyao Liu[1], Zhixiang Hu[1], Yuzhu Zhang[1], Hua-Yao Li[1], Naibo Gao[1], Zhilai Tian[1], Licheng Zhou[1], Baohui Zhang[1], Jiang Tang[1], Jianbing Zhang[1], Fei Yi[1], Huan Liu[1, *]

[1]School of Optical and Electronic Information, Wuhan National Laboratory for Optoelectronics, Huazhong University of Science and Technology, 1037 Luoyu Road, Wuhan 430074, People's Republic of China

*Corresponding author. E-mail: huan@hust.edu.cn (Huan Liu)

Supplementary Figures

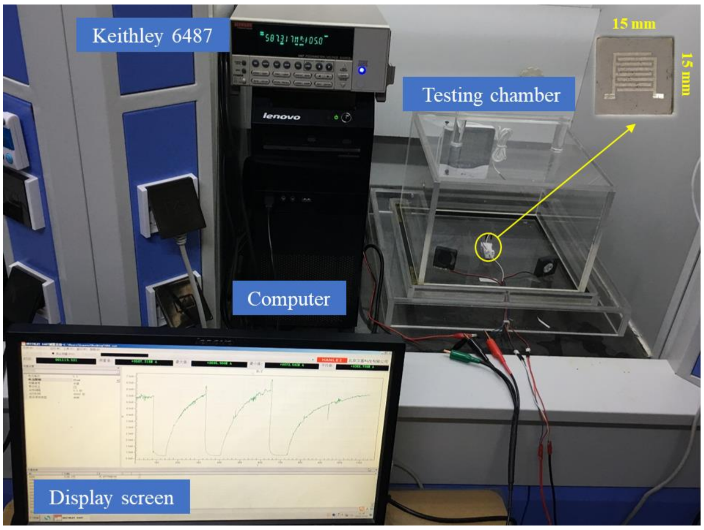

Fig. S1 Home-made sensor set-up for NO_2-sensing measurement under static conditions

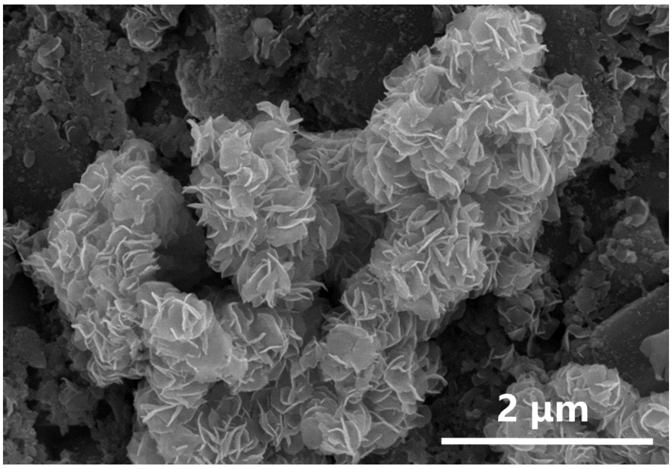

Fig. S2 SEM image of the flower-like MoS_2 nanosheets prepared on the alumina ceramic substrate

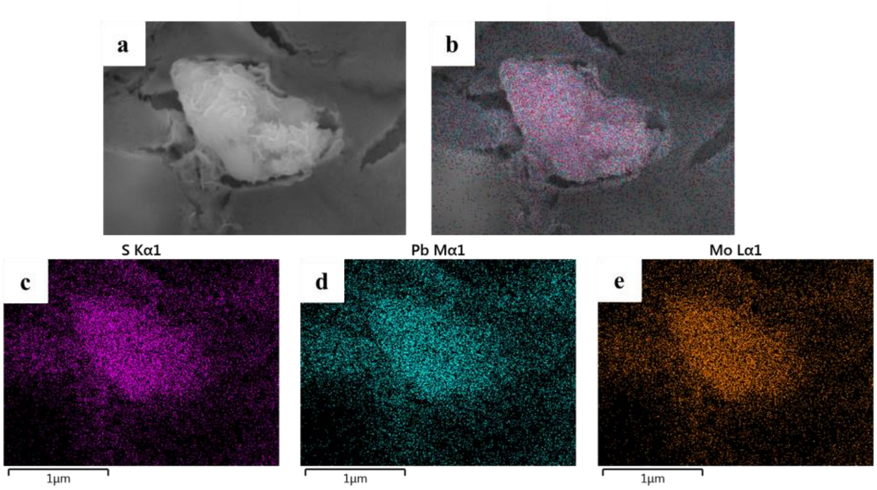

Fig. S3 (**a**) Typical elemental mapping of MoS_2 nanosheets sensitized with QDs: (**b**) Overlap, (**c**) S, (**d**) Pb, and (**e**) Mo

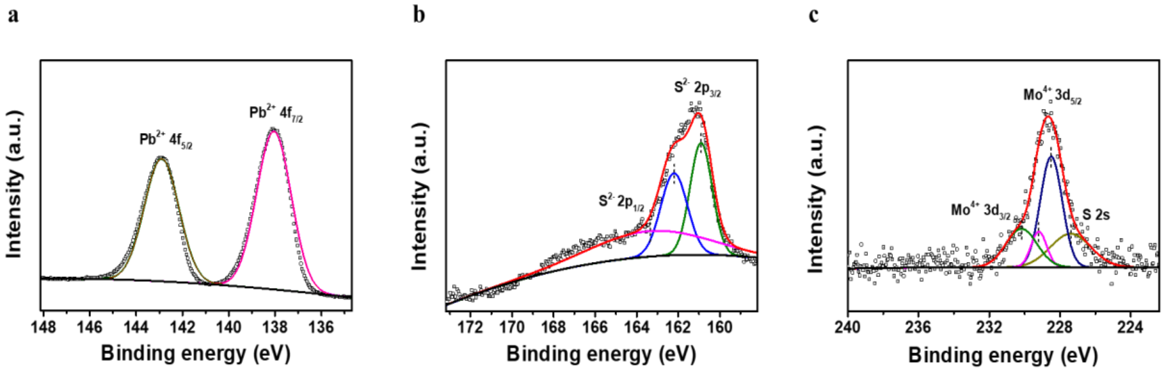

Fig. S4 X-ray photoelectron spectroscopy of MoS_2 nanosheets sensitized with QDs: (**a**) Pb 4f, (**b**) S 2p, and (**c**) Mo 3d elements corresponding to Pb^{2+}, S^{2-}, and Mo^{4+}

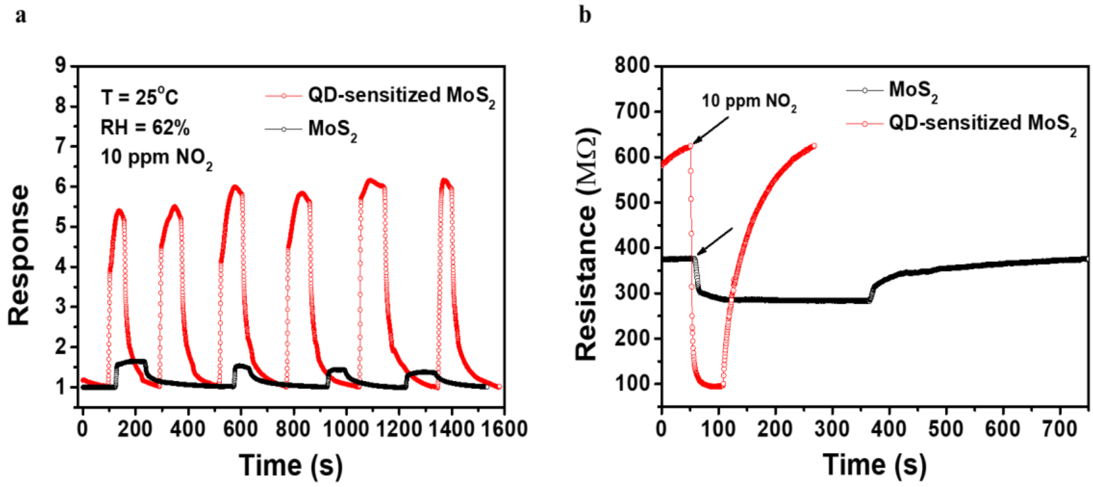

Fig. S5 (**a**) Repeatability curves of the MoS_2 nanosheets and the sensitized MoS_2 gas sensors exposed to 10 ppm concentration at room temperature, respectively. (**b**) Transient resistance characteristic of MoS_2 nanosheets and the sensitized MoS_2 gas sensors to 10 ppm NO_2

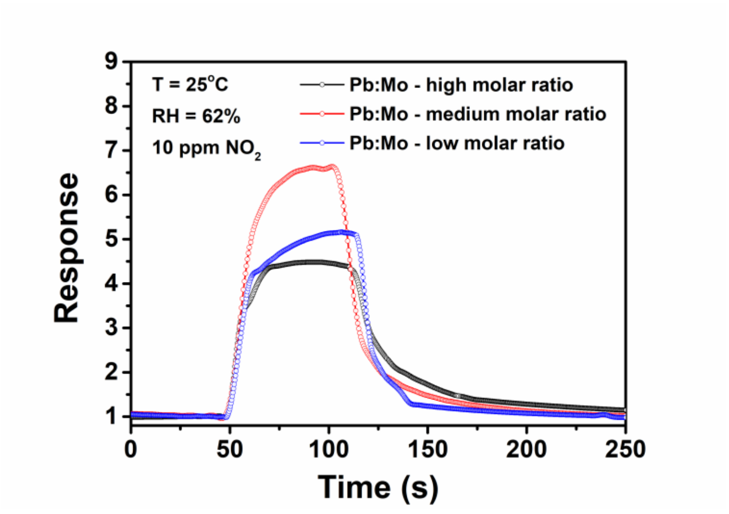

Fig. S6 Sensor response of the fabricated device using QD-sensitized MoS_2 with different molar ratio of Pb to Mo

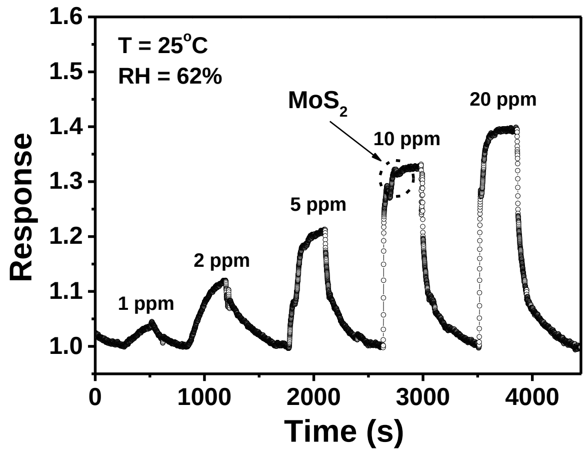

Fig. S7 Transient relative response of MoS_2 sensors toward different NO_2 concentrations

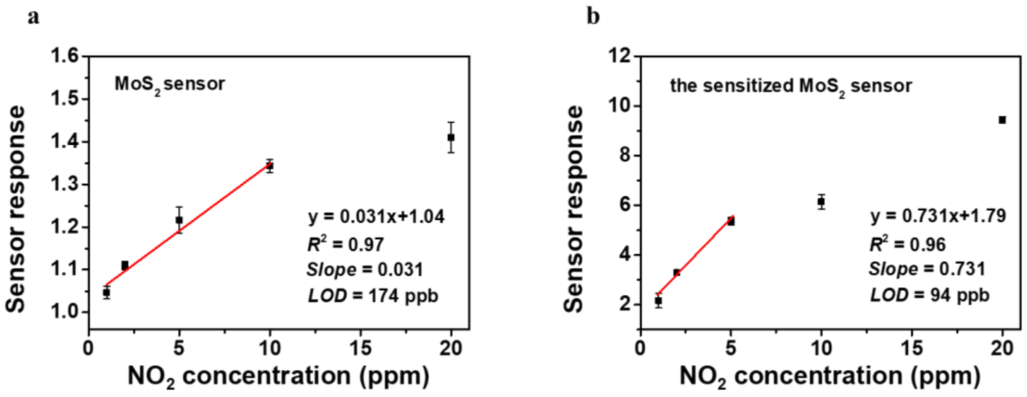

Fig. S8 Calculation of the limit of detection (*LOD*). The linear fitting of (**a**) the MoS_2 sensor response and (**b**) the sensitized MoS_2 sensor response with NO_2 concentrations in the linear region, the *slope* was 0.031 and 0.731 ppm^{-1}, respectively. We replotted 150 data points at the baseline before the NO_2 exposure and calculated the RMS_{noise} to be 0.018 and 0.023, respectively. According to *LOD* calculation equation *LOD* (ppm) = $3 \times RMS_{noise}/Slope$, the *LOD* was 174 and 94 ppb for the pristine MoS_2 and the sensitized MoS_2 sensors

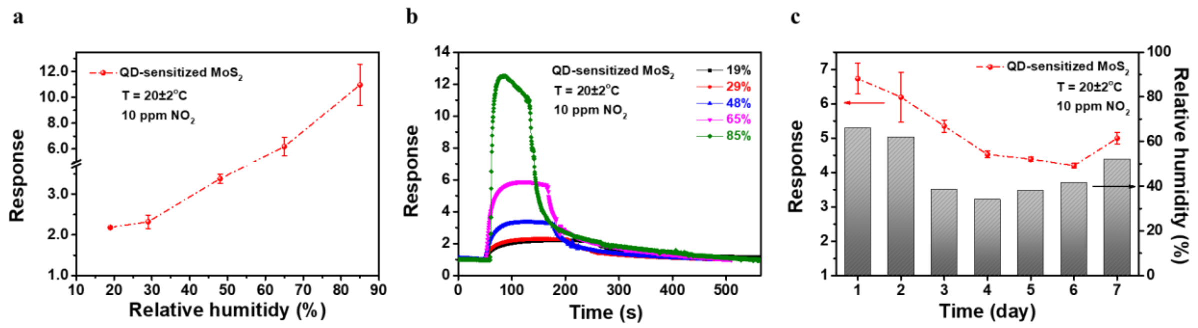

Fig. S9 (**a**) Sensor response toward 10 ppm NO_2 at different relative humidity based on the QD-sensitized MoS_2 gas sensors. (**b**) Real-time sensing curves toward 10 ppm NO_2 at different relative humidity based on the QD-sensitized MoS_2 gas sensors. (**c**) Long-term stability of the QD-sensitized MoS_2 gas sensors toward 10 ppm NO_2

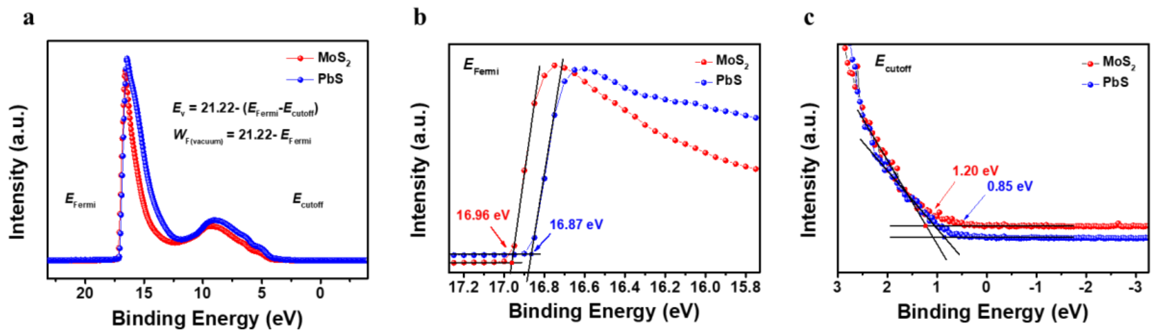

Fig. S10 Ultraviolet photoelectron spectroscopy of MoS_2 nanosheet and PbS QD. (**a**) shows the full scan of the spectra. From the partial data of (**b**) showing 16.96 eV and 16.87 eV for the secondary electron onset for MoS_2 and PbS, respectively (considering the He energy of 21.22 eV), we calculated the Fermi Energy positions ($W_{F\text{-vacuum}}$) relative to a vacuum of 4.26 eV for MoS_2 and 4.35 eV for PbS. (**c**) The corresponding fits to the valence band maximum (VBM-E_v); the cut-off gave VBMs of 5.46 eV for MoS_2 and 5.20 eV for PbS

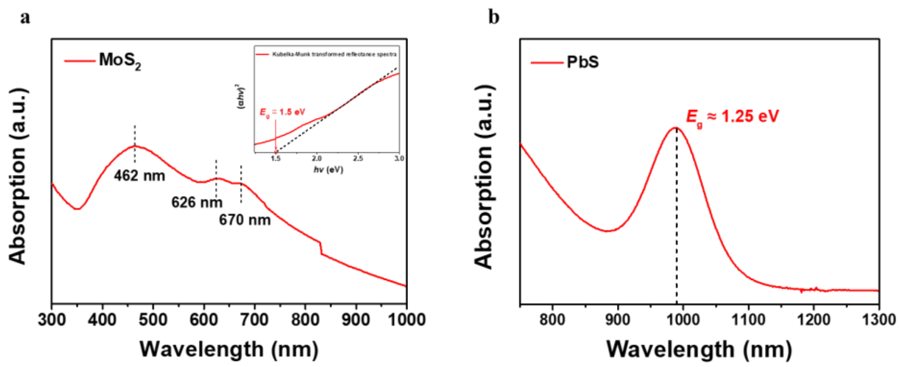

Fig. S11 UV-Vis-NIR spectra of MoS_2 nanosheet and PbS QD. (**a**) The characteristic absorption peaks appeared in visible regions were consistent with the general features of TMDs with trigonal prismatic coordination, which confirmed the 2H polytype of MoS_2 nanosheet. The intercept was interpolated in (a) of $(\alpha h\nu)^2$ as a function of photon energy hν showing the intersection between the linear fit and the photon energy axis gives the value to energy band gap (E_g) of 1.5 eV for MoS_2. (**b**) The excitonic peak in 992 nm confirming conservation of strong quantum confinement in PbS QD, and the calculative E_g was 1.25 eV according to the formula E_g=1240/ λ

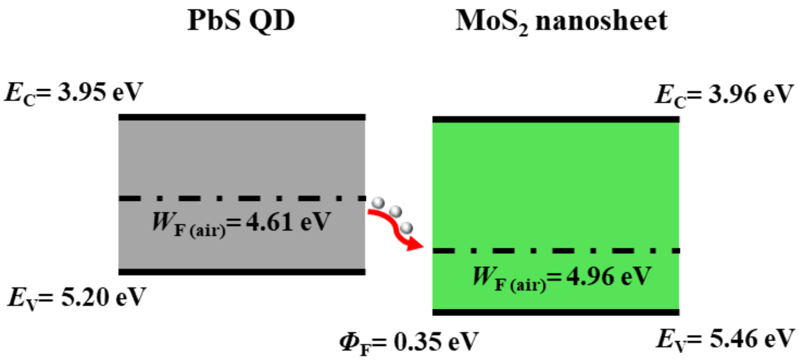

Fig. S12 Initial energy band structure of PbS QD and MoS_2 nanosheet before mutual contact. E_F denotes the Fermi level, W_F is the work function in the ambient air, and E_C and E_V are the conduction band edge and valence band edge

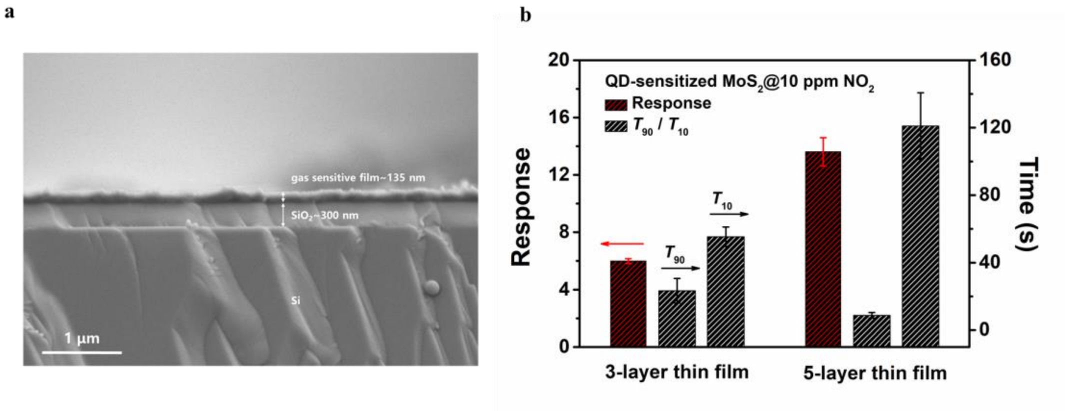

Fig. S13 (**a**) SEM cross-section morphology of the 3-layer QD-sensitized MoS_2 thin film. (**b**) NO_2-sensing properties of the fabricated sensors using QD-sensitized MoS_2 with different deposition layers
